# Metastases of Renal Cell Carcinoma to the Thyroid Gland with Synchronous Benign and Malignant Follicular Cell-Derived Neoplasms

**DOI:** 10.1155/2013/485025

**Published:** 2013-04-11

**Authors:** Carlos Zamarrón, Ihab Abdulkader, María C. Areses, Vanesa García-Paz, Luís León, José Cameselle-Teijeiro

**Affiliations:** ^1^Department of Respiratory Medicine, Clinical University Hospital, SERGAS, Health Research Institute of Santiago de Compostela (IDIS), University of Santiago de Compostela, 15706 Santiago de Compostela, Spain; ^2^Department of Anatomic Pathology, Clinical University Hospital, SERGAS, Health Research Institute of Santiago de Compostela (IDIS), University of Santiago de Compostela, 15706 Santiago de Compostela, Spain; ^3^Department of Medical Oncology, University Hospital Complex of Ourense, SERGAS, 32005 Ourense, Spain; ^4^Department of Medical Oncology, Clinical University Hospital, SERGAS, Health Research Institute of Santiago de Compostela (IDIS), University of Santiago de Compostela, 15706 Santiago de Compostela, Spain

## Abstract

Clear cell renal cell carcinoma (CCRCC) is the most common origin for metastasis in the thyroid. A 51-year-old woman was referred to our hospital for a subcarinal lesion. Ten years before, the patient had undergone a nephrectomy for CCRCC. Whole-body fluorodeoxyglucose positron emission tomography revealed elevated values in the thyroid gland, while the mediastinum was normal. An endoscopic ultrasonography-guided fine-needle aspiration biopsy of the mediastinal mass was consistent with CCRCC, and this was confirmed after resection. The thyroidectomy specimen also revealed lymphocytic thyroiditis, nodular hyperplasia, one follicular adenoma, two papillary microcarcinomas, and six foci of metastatic CCRCC involving both thyroid lobes. Curiously two of the six metastatic foci were located inside two adenomatoid nodules (tumor-in-tumor). The metastatic cells were positive for cytokeratins, CD10, epidermal growth factor receptor, and vascular endothelial growth factor receptor 2. No *BRAF* gene mutations were found in any of the primary and metastatic lesions. The patient was treated with sunitinib and finally died due to CCRCC distant metastases 6 years after the thyroidectomy. In CCRCC patients, a particularly prolonged survival rate may be achieved with the appropriate therapy, in contrast to the ominous prognosis typically found in patients with thyroid metastases from other origins.

## 1. Introduction

Renal cancer represents around 3% of adult malignancies [1]. Clear cell renal cell carcinoma (CCRCC) is the most frequent histological type, and although the clinical course of CCRCC is unpredictable, there is a high probability of late distant metastasis occurring in unusual locations [1–3]. In fact after radical nephrectomy, long-term monitoring is necessary because CCRCC may recur many years after diagnosis [3]. CCRCC remains one of the most lethal of the major genitourinary malignancies with 30% to 40% of patients eventually dying from the disease [4]. About 20–30% of patients with CCRCC have synchronous distant metastases at the time of primary diagnosis; another 30% develop metachronous metastases [2]. In autopsy series, thyroid metastases from CCRCC have been detected in 4-5% of patients, but those that are clinically significant are a rare event [2]. 

In earlier studies cytokine therapy with interferon-alpha or interleukin-2 (IL-2) was shown to induce objective responses, although only achieving durable survival rates in a minority of patients with renal cell carcinoma [5]. Recent advances in understanding the molecular biology of common CCRCC have resulted in the development of drugs that target known molecular pathways involved in cellular proliferation and neoangiogenesis. Agents, such as axitinib, sunitinib, bevacizumab, sorafenib, and temsirolimus, have been used in several clinical settings in the management of metastatic or recurrent CCRCC [6].

Here, we present a case of recurrent CCRCC, with metastases to both the mediastinal lymph nodes and the thyroid gland 10 years after nephrectomy. Curiously, a peculiar distribution of metastatic foci inside adenomatoid nodules (tumor-in-tumor) was found. In addition, a particularly prolonged survival rate was achieved, in contrast to the ominous prognosis typically found in patients with thyroid metastases from other origins. 

## 2. Case Presentation

### 2.1. Clinical Summary

**Figure 1:**
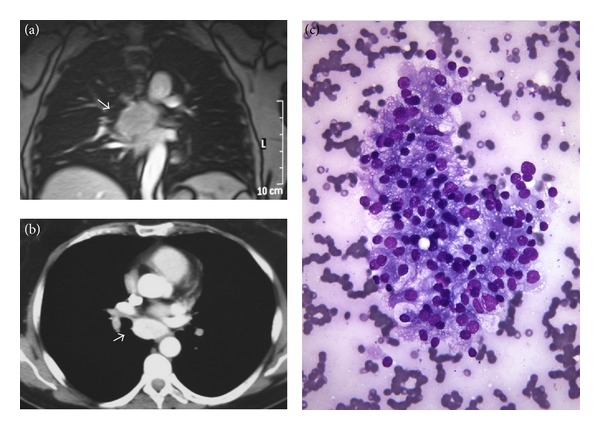
Metastatic renal cell carcinoma. (a and b) Computed tomography showed the renal metastatic mass (arrow) in the mediastinum. (c) Fine-needle aspiration biopsy guided by endoscopic ultrasonography of the mediastinal tumor revealed clusters of epithelial malignant cells morphologically fitting with metastatic clear cell renal cell carcinoma (Diff-Quick).

A 51-year-old Caucasian woman presented with a 4 cm well-defined hyperdense subcarinal mass detected during a surveillance computed tomography (CT) scan (Figures 1(a) and 1(b)). Ten years before, the patient had been diagnosed as having a CCRCC (nuclear grade 2, stage pT1bN0M0) and underwent a left nephrectomy with an adjuvant therapy of IL-2, subcutaneously, in a clinical trial. There was no history of von Hippel-Lindau syndrome or other hereditary cancer disorders.

A bronchoscopy showed enlargement of the carina, and a fine-needle aspiration biopsy (FNAB) guided by endoscopic ultrasonography revealed clusters of epithelial malignant cells with clear cytoplasm morphologically fitting with metastatic CCRCC (Figure 1(c)). The whole-body fluorodeoxyglucose positron emission tomography (FDG-PET) was negative in the mediastinum, but a higher value uptake in the thyroid gland was present. The patient underwent thoracotomy with complete excision of the mediastinal lesion; the pathological examination revealed a metastatic CCRCC. A thyroid gammagraphy showed two cold nodules in a multinodular goiter, and a thyroidectomy was performed in order to rule out a primary thyroid neoplasm.

One year after the thyroidectomy, a new CT scan showed multiple pulmonary nodules, a right supraclavicular adenopathy, and metastasis in the left rhomboid muscle. Treatment was started with sunitinib 50 mg/day, and the patient remained clinically stable for a 5-year-period but finally died due to multiple metastases 6 years after the thyroidectomy.

### 2.2. Pathological Findings

**Figure 2:**
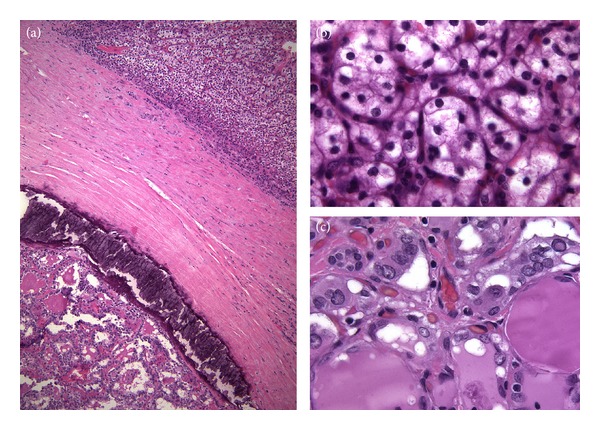
Concurrent renal cell carcinoma and thyroid carcinoma. (a) Thyroidectomy specimen showed synchronous foci of metastatic clear cell renal cell carcinoma (top) and papillary thyroid carcinoma (bottom) (hematoxylin and eosin). The characteristic clear cell cytoplasm of the renal cell carcinoma (b) and the peculiar nuclear features of papillary thyroid carcinoma (c) are seen at a higher magnification (hematoxylin and eosin).

**Figure 3:**
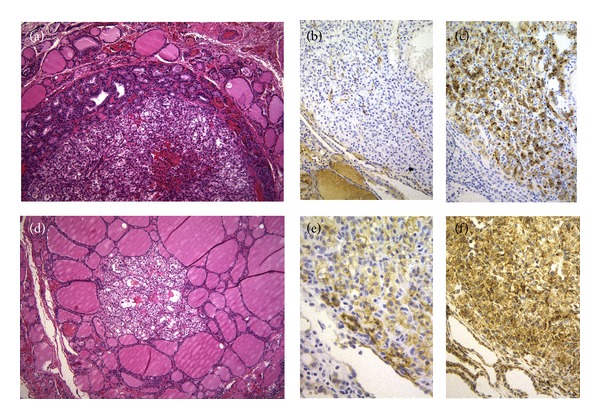
Tumor-in-tumor. (a and d) Two foci of metastatic renal cell carcinoma presented as central lesions inside hyperplastic adenomatoid nodules (hematoxylin and eosin). Tumor cells of renal cell carcinoma were negative for thyroglobulin (b) but positive for CD10 (c), EGFR (e), and VEGFR2 (f).

Histologic examination of the thyroidectomy specimen showed a lymphocytic thyroiditis, nodular hyperplasia (multinodular goiter), one follicular adenoma, two foci of papillary microcarcinoma, follicular variant, measuring 2 and 4 mm, and six foci of metastatic CCRCC, measuring from 1 to 8 mm in diameter, involving both thyroid lobes (Figures 2 and 3). Curiously, two foci of CCRCC presented as central lesions inside hyperplastic adenomatoid nodules of follicular cells (Figures 3(a)–3(f)). In the immunohistochemical study performed on paraffin sections using a peroxidase-conjugated labeled dextran polymer (Dako EnVision Peroxidase/DAB; Dako, Glostrup, Denmark), the papillary microcarcinomas of the thyroid were positive for cytokeratins (clone AE1/AE3, ready-to-use, Dako), thyroglobulin (8G7G3/1, dilution 1 : 20; Dako), thyroid transcription factor-1 (TTF-1, 8G7G3/1, ready-to-use, Dako), and Hector Battifora mesothelial cell (HBME-1, dilution 1 : 200; Dako), with negativity for calcitonin (polyclonal, dilution 1 : 5,000; BioGenex, San Ramon, CA); renal tumor cells showed reactivity for cytokeratins, CD10 (56C6, ready-to-use, Dako) (Figure 3(c)), epidermal growth factor receptor (EGFR, EGFR pharmDx, Dako) (Figure 3(e)), and vascular endothelial growth factor receptor 2 (VEGFR2) (FLK1, 1 : 2000, Santa Cruz, CA) (Figure 3(f)), but no immunoreaction was found for thyroglobulin (Figure 3(b)), TTF-1, HBME-1, vascular endothelial growth factor (VEGF) (VG1, 1 : 20, Dako), VEGFR1 (FLT1, 1 : 1000, Santa Cruz), and VEGFR3 (FLT4, 1 : 19, Novocastra, Newcastle upon Tyne, England).

We also screened for mutations in exon 15 of the *BRAF* gene as previously described [7], but no mutations were found in the tissue samples from the hyperplastic thyroid tissue, follicular carcinoma, papillary microcarcinomas, or metastatic CCRCC.

## 3. Discussion

Metastases from nonthyroid malignancies to the thyroid gland have been reported in 1.4%–3% of all patients who have surgery for thyroid malignancy, whereas the autopsy studies report a wide range of prevalences, from 1.9% to 24%, for these metastases to the thyroid [2]. CCRCC has also been known to present as a metastatic carcinoma of unknown primary, sometimes discovered in unusual sites [1, 8] but should be considered in the differential diagnosis of a thyroid nodule, particularly in patients who have a history of malignancies [9, 10]. In the present case, the histopathological examination of the thyroid showed chronic thyroiditis, multinodular hyperplasia, one follicular adenoma, two papillary microcarcinomas, and multiple foci of metastatic CCRCC. 

Classically, in clinical series, the most common primary sites of metastasis in the thyroid were kidney, lung, uterus, and melanoma; however, breast, lung, and skin (melanoma) were the most common sites of metastases origin in autopsy series [9–12]. While in autopsy series, metastases to the thyroid were most often multifocal and variable in size, in clinical series they were more commonly solitary and could measure up to 15 cm in diameter [11]. Based on a recent review of the literature [2], however, the most common nonthyroid malignancies that metastasize to the thyroid gland are renal cell (48.1%), colorectal (10.4%), lung (8.3%), breast carcinomas (7.8%), and sarcomas (4.0%). Curiously, CCRCC can present as a thyroid mass in the absence of renal symptoms years or decades after the removal of the primary tumor [2, 9]. As occurred in our case, metastases to the thyroid are more common in women than men (female to male ratio, 1.4 : 1) and in nodular thyroid glands (44.2%) [2]. 

Although metastasis of “cancer to cancer” (“tumor-to-tumor,” “tumor-in-tumor,” and “one to another”) is a well-established phenomenon, occurrence implicating a renal cancer is rare [13]. Tumor-to-tumor metastasis in thyroid neoplasms is exceedingly uncommon [14, 15]. In the present case, the identification of metastatic renal cell carcinoma was confirmed by the absence of thyroglobulin and TTF-1 in the neoplastic cells and by the positivity for CD10.

Several studies have indicated that abnormal glands (nodular goiter, adenomas, well-differentiated carcinomas, thyroiditis, etc.) are more likely to harbor metastatic disease than normal glands, presumably due to abnormal blood supply resulting in decreased oxygen and iodine content [2, 9, 11]. In rare instances, tumor-in-tumor phenomena reflect tumor-to-tumor metastases, mainly to follicular adenoma or to follicular variant of papillary thyroid carcinoma [14]. Primary papillary thyroid carcinoma can also grow in the core of a follicular adenoma exhibiting a tumor-in-tumor pattern [16]. An encapsulated thyroid tumor having 3 different concentric appearances has also been reported recently as another expression of tumor-in-tumor in the thyroid gland [17]. In our case, metastatic CCRCC coexisted with papillary thyroid microcarcinomas, and interestingly, two foci of metastatic CCRCC were located inside hyperplastic thyroid nodules, suggesting that the microenvironment in the thyroid nodules is particularly attractive for renal carcinoma tumor cells.

FNAB of thyroid masses is useful in diagnosis of thyroid metastases; this, however, requires information about the nonthyroid malignancies, so that proper antibodies can be used for immunohistochemical studies [2]. False negatives with FDG-PET can be explained because some renal tumors have a low growth rate with low glucose metabolism, sometimes with differences in the glucose uptake of several metastases in the same tumo or even due to tumor necrosis. The discordant PET that results in the present case may be related to a different pattern in the metastasis or to follicular cell-derived lesions. To improve the sensitivity and specificity of FDG-PET in renal cancer, new radiotracers such as choline are being developed [18].

In our patient, mediastinal metastasectomy was carried out because the PET showed no tracer uptake elsewhere. A particularly favorable long-term outcome in selected patients after metastasectomy with CCRCC has been well documented in many studies, and an overall 5-year survival rate of 30–50% is common if isolated metastatic lesions are resected [19]. EGFR protein overexpression is frequent in renal cell carcinoma and correlates with the chromosome 7 polysomy and poor prognostic parameters in clear cell renal cell carcinoma [1, 20]. Sunitinib is an oral receptor tyrosine kinase inhibitor that targets signaling by PDGFRs, VEGFRs, and c-kit [21]. In our patient, treatment was started with sunitinib, and a prolonged survival rate was achieved.

As our case exemplifies, in CCRCC patients, a particularly prolonged survival rate may be achieved with the appropriate therapy, in contrast to the ominous prognosis typically found in patients with thyroid metastases from other origins. At the same time, the peculiar distribution of CCRCC foci inside adenomatoid nodules (tumor-in-tumor) suggests that the microenvironment in the thyroid nodules is particularly attractive for renal carcinoma tumor cells.
